# Observation of a Relationship Between Orbital-Specific Molecular Similarity Index and Toxicity of Methylcarbamate Derivatives

**DOI:** 10.3390/molecules30142947

**Published:** 2025-07-12

**Authors:** Sihan Long, Yuuki Onitsuka, Soichiro Nagao, Masahiko Takahashi

**Affiliations:** Institute of Multidisciplinary Research for Advanced Materials, Tohoku University, Sendai 980-8577, Japan; long.sihan.s7@dc.tohoku.ac.jp (S.L.); yuuki.onitsuka.e8@tohoku.ac.jp (Y.O.); soichiro.nagao.p7@dc.tohoku.ac.jp (S.N.)

**Keywords:** molecular similarity index, momentum-space wavefunction, frontier orbital theory, methylcarbamate derivatives

## Abstract

We report a computational investigation on the reachability of the molecular similarity index (MSI) approach for predicting the relative drug strength of methylcarbamate derivatives. Traditional MSI values have been obtained by calculating the overlap integral of total electron momentum densities between one molecule and another. Furthermore, we have proposed and tested orbital-specific MSI (OS-MSI) values, obtained by doing the same but with electron momentum densities of a selected molecular orbital (MO) such as the highest occupied MO (HOMO) and the lowest unoccupied MO (LUMO). In the calculations, a Boltzmann-weighted electron momentum density estimated by a theoretical probability distribution of rotamers was used, while the solvation effect was considered using the polarizable continuum model. It is shown that the traditional MSI values as well as the OS-MSI values for the HOMO do not have any correlation with experimental relative toxicity of the methylcarbamate derivatives. In contrast, it has been observed and found that the OS-MSI values for the LUMO exhibit a noticeable correlation with the experimental data. The reason behind this observation is discussed in relation to the drug reaction mechanism of the methylcarbamate derivatives.

## 1. Introduction

In drug discovery, measures of molecular similarity have emerged as valuable tools for comparing structural features and elucidating chemical reactivity [1,2,3]. Over the past decades, one of the well-known approaches in assessing molecular similarity relied on electronic structure, in which we quantitatively examine the overlap between electron densities of different molecules via quantum chemical calculations [4,5]. Such an approach usually analyzes electron densities in position space (*r*-space), but they are often sensitive to the exact positions of atomic nuclei, resulting in making contributions of the more chemically relevant outer valence electrons obscure [6]. To address these issues, Cooper and Allan introduced a new and novel concept, molecular similarity index (MSI), that assesses the similarity of total electron densities in momentum space (*p*-space) between different molecules [7]. Basically, in *p*-space, all the nuclei are located at the origin of the (*p_x_*, *p_y_*, *p_z_*) coordinate space, so one can calculate the overlap integral of electron momentum densities between one molecule and another, without any restrictions due to their structural difference in *r*-space. Note that the information about the equilibrium nuclear positions in *r*-space is contained solely in the phase factors of the *p*-space wavefunctions introduced by the Fourier transform [8]. In addition, since a small *p* part of a *p*-space wavefunction corresponds to a large *r* part of the corresponding *r*-space one, electron momentum density is expected to be sensitive to the behavior of electrons far from the nuclei that may be of central importance in physicochemical properties such as chemical reactivity and molecular recognition [9]. In fact, Cooper and Allan successfully showed that the MSI values can be used to rationalize HIV virology data for the families of phospholipids and even to make some successful predictions of active compounds [10,11,12]. They subsequently applied the MSI method to evaluate the hyperpolarizabilities of benzenes and related compounds by using electron momentum densities of frontier orbitals [12], the highest occupied molecular orbital (HOMO) and the lowest unoccupied molecular orbital (LUMO).

The above-mentioned correlation of MSI values with HIV virology data for families of phospholipids [10,11,12] indicates that the MSI approach may possess a potential ability to predict the relative drug strength of various kinds of compounds. In this respect, however, the pioneering MSI studies of Cooper and Allan mainly focused on anti-HIV activities, and, hence, the reachability of the MSI approach for predicting relative drug strength is still unknown. Under these circumstances, we have thoroughly tested the reachability of the MSI approach for methylcarbamate derivatives widely used as pesticides [13,14]. The reason why we chose the methylcarbamate derivatives as the first pilot systems to be studied lies in their drug reaction mechanism [15]. It is well known that the carbamate toxicity originates from inhibition to acetylcholinesterase (AChE) enzyme that isolates from insects and mammals, causing accumulation of acetylcholine and negative influences on neurotransmission [16,17]. Regarding this, a reaction mechanism model has been proposed [15], in which the electrophilic carbonyl carbon atom of a carbamate derivative reacts with a serine of the active site in the AChE enzyme. This means, from a standpoint of the frontier orbital theory [18], that the majority of energy gain in the drug reaction is a result of the reaction between the HOMO of a serine of the active site in the AChE enzyme (nucleophile) and the LUMO of a carbamate derivative (electrophile). One can therefore expect that traditional MSI values associated with total electron momentum density do not exhibit any correlation with experimental data of relative toxicity of the methylcarbamate derivatives [14], but orbital-specific MSI (OS-MSI) values associated with electron momentum density of the LUMO orbital may show some correlation. To the best of our knowledge, there have been no MSI studies on this aspect and this is exactly the central issue of the present study that aims to test the potential ability of the MSI approach to predict relative drug strength.

In the present study, we report a computational investigation on the reachability of the MSI approach for predicting the relative drug strength of the methylcarbamate derivatives. In the calculations, we have introduced three upgrades that were not employed in the pioneering MSI studies of Cooper and Allan. Firstly, we propose and test OS-MSI values in addition to testing traditional MSI ones, as explained above. Secondly, we consider not only the equilibrium structure of a molecule but also its rotamers. A Boltzmann-weighted electron momentum density is calculated using a theoretical probability distribution of rotamers. Lastly, we consider the solvation effect on electron momentum density, as drugs usually react with their targets in solution. It is well known that solvation effect has a great influence in the majority of biological processes and even a minor effect on the molecular electronic structures of drugs may largely affect their biological functionality [19]. This paper is organized as follows. Section 2 describes the methylcarbamate derivatives studied, as well as the traditional MSI and newly proposed OS-MSI methods and details of theoretical calculations made for the methylcarbamate derivatives. In Section 3, the results of the calculations are presented and discussed in relation to the reaction mechanism. Section 4 contains a summary of the present study and some current progress.

## 2. Materials and Methods

### 2.1. Methylcarbamate Derivatives and Their Toxicity

**Figure 1:**
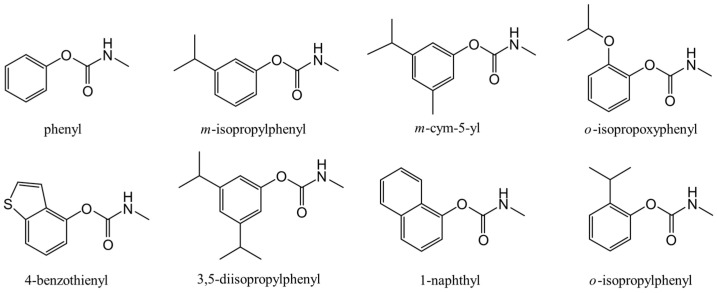
Structural models of phenyl methylcarbamate and its derivatives.

Carbamates are derivatives of carbamic acid and are identified by the presence of the -O-CO-N- linkage. Carbamates are an integral part of many drugs and many compounds that contain a carbamate group are currently in various stages of preclinical and clinical trials [13]. Figure 1 shows structural models of the methylcarbamate derivatives that we chose for the present study. They are phenyl-, *m*-isopropylphenyl-, *m*-cym-5-yl-, *o*-isopropoxyphenyl-, 4-benzothienyl-, 3,5-diisopropylphenyl-, 1-naphthyl-, and *o*-isopropylphenyl-methylcarbamates. It can be seen from the figure that in this methylcarbamate group, phenyl methylcarbamate has the platform skeleton and all others are derivatives of this molecule.

Experimental LD_50_ (mg/kg) values of the methylcarbamate derivatives for rats are taken from the literature [14] and are listed in Table 1 as values in mol/kg. LD_50_ is the amount of a toxic agent that kills 50% (one half) of a population of test animals, so its reciprocal represents the drug strength of a compound. Here the experimental LD_50_ data are listed by three routes of administration (intravenous, intraperitoneal, and oral injections), and they are different from each other due to the difference in the kinetic process of toxicity testing. Among those, the intravenous injection data may be best suited to be compared with MSI and OS-MSI values. This is because the MSI approach is wholly based on the molecular electronic structure and the intravenous injection is the most direct route without influences by environmental factors such as absorption and elimination of compounds in the intermediate processes of other routes [20]. It can be seen from Table 1 that different substitutions at the phenyl termini of phenyl methylcarbamate provide different changes in LD_50_ values of the intravenous injection data. Some substitutions enhance the LD_50_ value and some reduce. Such a trend is difficult to understand by considering the molecular structures of the methylcarbamate derivatives alone. It is the main purpose of this paper to test the ability of the MSI approach to predict the trend regarding their relative drug strength.

### 2.2. Definition of MSI and OS-MSI

In the traditional MSI method of Cooper and Allan [7,10,11,12], $I_{std,i}^{MSI}\left(n\right)$ is used as a measure of similarity between one molecule and another, which is defined as

(1) $$I_{std,i}^{MSI}\left(n\right)=\frac{2\int p^{n}\left[\sum_{j}\rho_{std}\left(p_{j}\right)\right]\left[\sum_{k}\rho_{i}\left(p_{k}\right)\right]dp}{\int p^{n}{\left[\sum_{j}\rho_{std}\left(p_{j}\right)\right]}^{2}dp+\int p^{n}{\left[\sum_{k}\rho_{i}\left(p_{k}\right)\right]}^{2}dp}\;.\;\;\;$$

Here $\sum_{j}\rho_{std}\left(p_{j}\right)$ and $\sum_{k}\rho_{i}\left(p_{k}\right)$ are total electron momentum densities of a standard reference molecule (*std*) and a test molecule (*i*), respectively. Total electron momentum density is calculated by summing up electron momentum density over all occupied electrons (*j* or *k*). Powers of *p*, $p^{n}$, can be used to emphasize different regions of momentum space, while the *n* value of zero is employed in the present study to highlight low momentum components of electrons. $I_{std,i}^{MSI}\left(n=0\right)$, which is hereafter referred to as $I_{std,i}^{MSI}$ can have a value between zero and unity. The $I_{std,i}^{MSI}$ value of unity means that $\sum_{j}\rho_{std}\left(p_{j}\right)$ and $\sum_{k}\rho_{i}\left(p_{k}\right)$ have completely the same total electron momentum density in the (*p_x_*, *p_y_*, *p_z_*) coordinate space. The $I_{std,i}^{MSI}$ value can thus be recognized as an index of how much $\sum_{k}\rho_{i}\left(p_{k}\right)$ of the test molecule is similar to $\sum_{j}\rho_{std}\left(p_{j}\right)$ of the standard reference molecule. On the other hand, in the OS-MSI method that we propose here, $I_{std,i}^{OSMSI}$ is used as a measure of similarity and it is given as follows:

(2) $$I_{std,i}^{OSMSI}=\frac{2\int p^{n}\rho_{std}\left(p_{j}\right)\rho_{i}\left(p_{k}\right)dp}{\int p^{n}{\left[\rho_{std}\left(p_{j}\right)\right]}^{2}dp+\int p^{n}{\left[\rho_{i}\left(p_{k}\right)\right]}^{2}dp}\;.\;$$

The difference between $I_{std,i}^{MSI}$ and $I_{std,i}^{OSMSI}$ is simply whether one considers the momentum density of all occupied electrons or one specific electron k in a molecule.

Basically, $\rho \left(p_{k}\right)$ is the absolute square of a *p*-space MO wavefunction $\psi_{k}\left(p\right)$, and $\psi_{k}\left(p\right)$ is uniquely related to the corresponding *r*-space MO wavefunction $\psi_{k}\left(r\right)\;$ through the Fourier transform:

(3) $$\psi_{k}\left(p\right)={\left(2\pi \right)}^{-\frac{3}{2}}\int drexp\left(-ip\cdot r\right)\psi_{k}\left(r\right).$$

Hence, it is possible for one to obtain $\rho \left(p_{k}\right)$ by taking the Fourier transform of $\psi_{k}\left(r\right)$ calculated, for instance, using a quantum chemistry calculation software such as Gaussian 16W package [21], and by taking the absolute square of the resulting $\psi_{k}\left(p\right)$. Details of the Fourier transform can be found in Ref. [22] and Supplementary Materials. $I_{std,i}^{MSI}$ and $I_{std,i}^{OSMSI}$ values are then calculated according to Equations (1) and (2), respectively.

### 2.3. Theoretical Calculations

To identify the reachability of the MSI approach most closely, calculations of $I_{std,i}^{MSI}$ and $I_{std,i}^{OSMSI}$ values for the methylcarbamate derivatives were carried out in the following manner. Firstly, we investigated the stable molecular structure of phenyl methylcarbamate in water solvent, because the molecule is the platform skeleton for other methylcarbamate derivatives. Full and partial geometry optimization calculations were made by using the Gaussian 16 package with the B3LYP/6-31G** density functional theory [21]. In the calculations, the polarizable continuum model (PCM) method [23] was employed. The calculations were also performed for the molecule in gas phase as a reference. Secondly, similar calculations were repeated for other methylcarbamate derivatives. As a result, we obtained rotamers for the individual molecules in water solvent at the rat body temperature of 310.25 K [24], which are to be considered in calculating $I_{std,i}^{MSI}$ and $I_{std,i}^{OSMSI}$ values. Thirdly, MOs were generated at the equilibrium geometries of each rotamer to be considered by using the B3LYP/6-31G** density functional theory with the PCM method. Lastly, $I_{std,i}^{MSI}$ and $I_{std,i}^{OSMSI}$ values were calculated according to Equations (1) and (2). Here we chose *m*-isopropylphenyl methylcarbamate as the standard reference molecule, as its drug strength is the largest among the molecules studied in the present study. The calculations of $I_{std,i}^{MSI}$ and $I_{std,i}^{OSMSI}$ values were performed by using a home-made program developed for the present study. Here, the -C-C-O- linkage of the phenyl group was aligned so that all the MOs of the methylcarbamate derivatives share the common (*p_x_*, *p_y_*, *p_z_*) coordinate space.

## 3. Results and Discussion

### 3.1. Conformers and Rotamers of Solvated Methylcarbamate Derivatives

**Figure 2:**
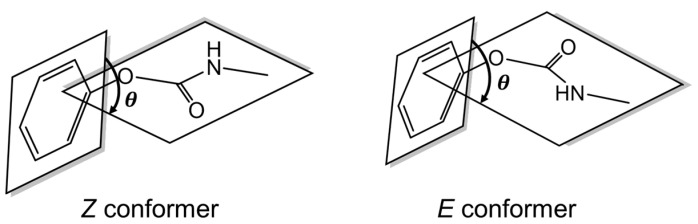
Schematic examples of *Z* and *E* conformers of phenyl methylcarbamate with a dihedral angle *θ*.

We begin discussion with *Z* and *E* conformers of phenyl methylcarbamate. For ease of later discussion, we define a C-C-O-C dihedral angle *θ* here, which is the angle between the benzene ring plane and the plane formed by carbamic acid skeleton. Examples of the *Z* and *E* conformers with a dihedral angle *θ* are shown in Figure 2. The full geometry optimization calculations at the DFT B3LYP/6-31G** level indicate that the *Z* conformer is more stable than the *E* conformer by 1506 and 949 cm^−1^ in gas phase and water solvent, respectively. The gas phase result is in accord with the study on isolated (gas phase) methyl carbamate using experimental infrared spectroscopy and theoretical calculations with the modified neglect of diatomic overlap (MNDO) method [25]. Furthermore, the energy difference between the *Z* and *E* conformers in water solvent is substantially larger than the thermal energy (215.6 cm^−1^) at the rat body temperature of 310.25 K, so the former is clearly the main species. Similar observations have been made for other methylcarbamate derivatives. We thus consider only the *Z* conformers of the methylcarbamate derivatives throughout the present work.

**Figure 3:**
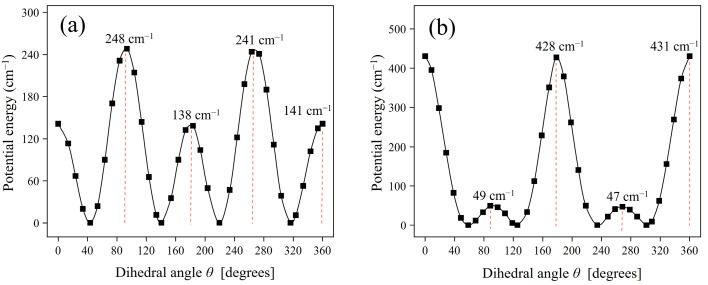
Torsional potential energy curves of the *Z* conformer of phenyl methylcarbamate in (**a**) gas phase and (**b**) water solvent.

Figure 3a,b show torsional potential energy curves of the *Z* conformer of phenyl methylcarbamate in gas phase and water solvent, respectively. The potential energy curves were obtained by the partial geometry optimization calculations, and they are plotted against the dihedral angle *θ* defined in Figure 2. A similarity and difference between the gas phase and water solvent data can be seen in Figure 3. The similarity is that rotamers of the *Z* conformer become the most stable at four dihedral angles both in gas phase and water solvent. On the other hand, the difference or solvation effect is that the four dihedral angles of the water solvent data take lower or higher angles (58.7°, 125.6°, 234.4°, and 301.3°) compared with the gas phase data (43.6°, 140.5°, 219.5°, and 316.4°). Furthermore, the difference lies also in potential barrier heights between adjacent rotamers. For instance, in gas phase (Figure 3a), high potential barriers of 240~250 cm^−1^ are at around *θ* = 93.6° and 263.6° and low barriers of about 140 cm^−1^ at around *θ* = 183.6° and 360.0°. However, in water solvent (Figure 3b), the high potential barriers in gas phase become low barriers of about 50 cm^−1^ at *θ* = 88.7° and 268.7° and vice versa. In addition, the two high barriers of about 430 cm^−1^ in water solvent are found to be larger than the thermal energy (215.6 cm^−1^) at the rat body temperature of 310.25 K, so no occurrence of conformational transitions over the barriers is reasonably assumed. However, the same is not true for the two low barriers of about 50 cm^−1^ in water solvent, as the values are smaller than the thermal energy. Conformational transitions over the low barriers have to be considered.

**Figure 4:**
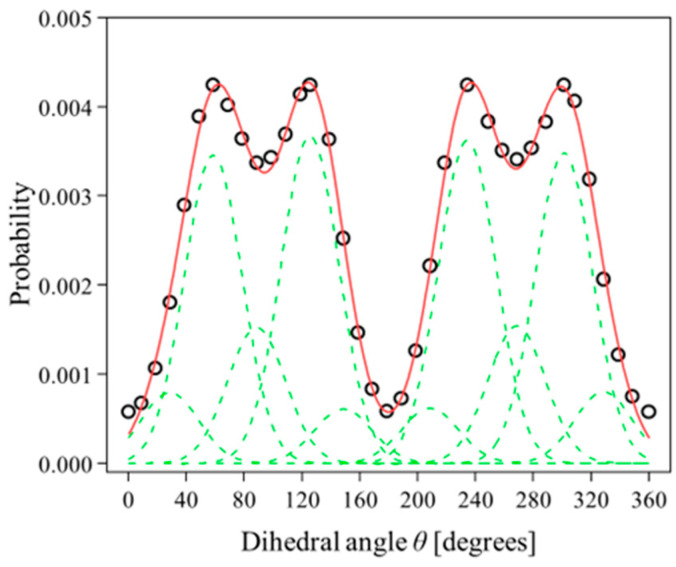
Curve fitting to an estimated probability distribution, shown by the open circles, of rotamers of phenyl methylcarbamate in water solvent over the entire dihedral angle θ range. The dashed and solid lines represent the deconvoluted curves and their sum, respectively. See the text for details.

We have thus estimated the probability distribution of rotamers over the entire dihedral angle *θ* range in the potential energy curve, by classically treating the Boltzmann distribution for rovibrational states of rotamers at the rat body temperature of 310.25 K. The results are shown by the open circles in Figure 4. A curve fitting to the estimated probability distribution has subsequently been attempted, by using ten Gaussian functions of the same width, centered at the four global maxima, two local minima of the probability distribution and four different dihedral angles. The results of the curve fitting are shown in Figure 4, with dashed lines for the deconvoluted curves and a solid line for their sum. It can be seen that the probability distribution is well reproduced by the curve fitting, showing that rotamers of phenyl methylcarbamate in water solvent can be approximated by a linear combination of ten rotamers with appropriate weight factors, which are relative intensities of the deconvoluted Gaussian functions obtained in the curve fitting.

The same data analysis as made for phenyl methylcarbamate has been repeated for other methylcarbamate derivatives in water solvent. We have found a similar behavior in terms of the tortional potential energy curve, barrier heights, and weight factors of rotamers to be considered for *m*-isopropylphenyl-, *m*-cym-5-yl-, and 3,5-diisopropylphenyl-methylcarbamates. For *o*-isopropoxyphenyl-, 4-benzotheinyl, 1-naphtyl-, and *o*-isopropylphenyl-methylcarbamates, however, only two global minima have been observed in their tortional potential energy curves and hence their weight factors of rotamers are rather different from the phenyl methylcarbamate case. The obtained weight factors for the methylcarbamate derivatives are summarized in Table 2. These weight factor values have been employed in the calculations of $I_{std,i}^{MSI}$ and $I_{std,i}^{OSMSI}$ values for the methylcarbamate derivatives.

### 3.2. Molecular Orbitals of Solvated Methylcarbamate Derivatives

**Figure 5:**
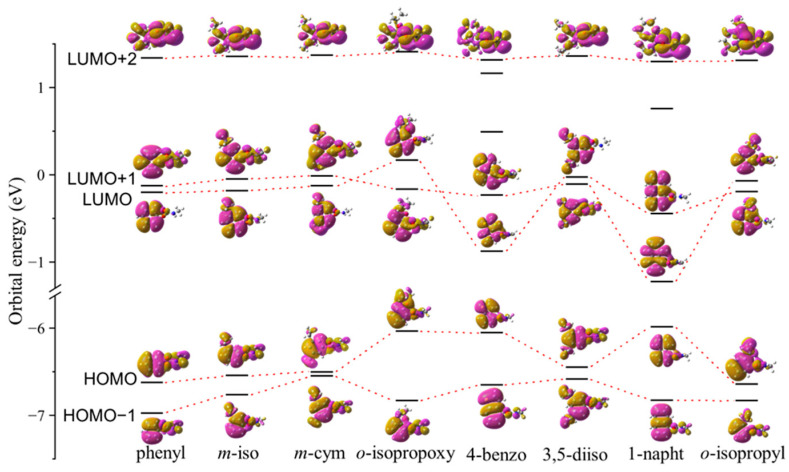
Molecular orbital correlation diagram of one of the most stable rotamers of methylcarbamate derivatives in water solvent. Phenyl, *m*-iso, *m*-cym, *o*-isopropoxy, 4-benzo, 3,5-diiso, 1-napht, and *o*-isopropyl indicate phenyl-, *m*-isopropylphenyl-, *m*-cym-5-yl-, *o*-isopropxyphenyl-, 4-benzothienyl-, 3,5-diisopropylphenyl-, 1-naphthyl-, and *o*-isopropylphenyl-methylcarbamates, respectively. The red dashed lines represent correlation of HOMO, HOMO−1, LUMO, LUMO+1, and LUMO+2 orbitals of the molecules. See the text for details.

Figure 5 shows orbital energies of the HOMO, next HOMO (HOMO−1), LUMO, next LUMO (LUMO+1), and second next LUMO (LUMO+2) of the methylcarbamate derivatives in water solvent, obtained by the DFT B3LYP/6-31G** calculations. Also included in the figure are insets that illustrate MO shapes in *r*-space, calculated for one of the most stable rotamers, which are those of phenyl-, *m*-isopropylphenyl-, *m*-cym-5-yl-, *o*-isopropoxylphenyl-, 4-benzothienyl-, 3,5-diisopropylphenyl-, 1-naphthyl-, and *o*-isopropylphenyl-methylcarbamates at the dihedral angles of 58.7°, 61.4°, 66.5°, 74.4°, 84.6°, 63.7°, 92.4°, and 92.2°, respectively. In the present study, since the order of theoretical MOs in terms of orbital energies sometimes interchanges [26,27], correlations among the individual MOs of the methylcarbamate derivatives were searched based on not the orbital energy but the MO shape similarity and they are shown by the dashed lines in Figure 5. Here designations such as LUMO and LUMO+1 are based on the order of the MOs of phenyl methylcarbamate in terms of orbital energies and the same designations are given to the correlated MOs of other methylcarbamate derivatives, as phenyl methylcarbamate has the platform skeleton of the other derivatives.

It can be seen from Figure 5 that different substitutions at phenyl termini of phenyl methylcarbamate provide different small changes in MO shape. The idea behind the OS-MSI method is that such a small change in shape of the frontier MO may lead to a large difference in drug strength and the $I_{std,i}^{OSMSI}$ values would be a good measure of similarity between the frontier MOs of two molecules, as will be discussed in the next subsection.

### 3.3. MSI and OS-MSI of Solvated Methylcarbamate Derivatives

**Figure 6:**
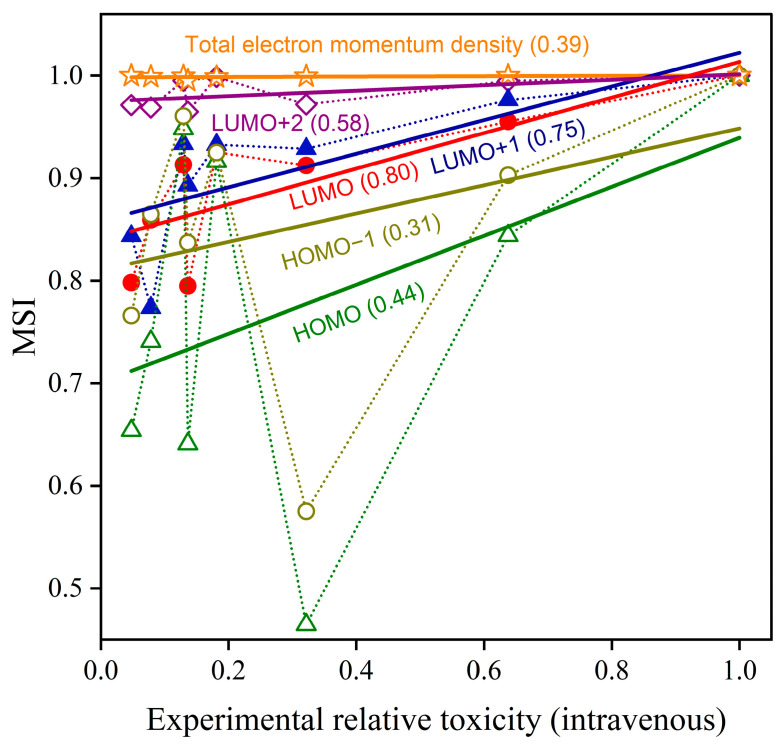
Plots of MSI values associated with total electron momentum density and OS-MSI values of the HOMO, HOMO−1, LUMO, LUMO+1, and LUMO+2 orbitals of methylcarbamate derivatives in water solvent against experimental relative toxicity values. The solid lines represent results of linear regression analysis and the number in parentheses represents the Pearson correlation coefficient value obtained for each data set. See the text for details.

Figure 6 plots the calculated $I_{std,i}^{OSMSI}$ values for the HOMO, HOMO−1, LUMO, LUMO+1, and LUMO+2 orbitals between a test molecule and the standard reference molecule (*m*-isopropylphenyl methylcarbamate) in water solvent against the experimental relative toxicity of the methylcarbamate derivatives. The experimental relative toxicity was obtained by dividing the reciprocal of the intravenous LD_50_ value of a test molecule in Table 1 by that of the standard reference molecule. On the other hand, the $I_{std,i}^{OSMSI}$ values have been obtained by summing up OS-MSI values $I_{std,i}^{OSMSI}(R,\;R^{\prime })$ over all pairs between a rotamer *R* of the standard reference molecule *std* and a rotamer *R*′ of a test molecule *i*, while considering their weight factors ($C_{std,R}$ and $C_{i,R^{\prime }}$) in Table 2:

(4) $$I_{std,i}^{OSMSI}=\frac{\sum_{R=1}^{10}\sum_{R^{\prime }=1}^{10}C_{std,R}C_{i,R^{\prime }}I_{std,i}^{OSMSI}(R,R^{\prime })}{\sum_{R=1}^{10}\sum_{R^{\prime }=1}^{10}C_{std,R}C_{std,R^{\prime }}I_{std,std}^{OSMSI}(R,R^{\prime })}$$

with

(5) $$\sum_{R}C_{std,R}=\sum_{R^{\prime }}C_{i,R^{\prime }}=1.\;$$

Here the summation has been taken over *R* and *R*′ from 1 to the number of rotamers to be considered, i.e., 10. We have also calculated the traditional $I_{std,i}^{MSI}$ values associated with total electron momentum density, but in a bit of a different way. This is because the number of bound electrons differs depending on the molecular size of the methylcarbamate derivative species and hence summing up the $I_{std,i}^{OSMSI}$ values over all the occupied electrons in a straightforward way makes the $I_{std,i}^{MSI}$ value size sensitive. We have therefore obtained $I_{std,i}^{MSI}$ in the following manner. Firstly, $\sum_{j}\rho_{std}\left(p_{j}\right)$ and $\sum_{k}\rho_{i}\left(p_{k}\right)$ calculated according to Equation (3) were each normalized by dividing them by the number of electrons in the standard reference molecule *std* and the test molecule *i*, respectively. The $I_{std,i}^{MSI}$ value was then calculated by summing up MSI values $I_{std,i}^{MSI}(R,\;R^{\prime })$ over all pairs between a rotamer *R* of the standard reference molecule *std* and a rotamer *R*′ of a test molecule *i*.

It is evident from Figure 6 that the ${\;I}_{std,i}^{MSI}$ values associated with total electron momentum density remain unaltered with the change in the test molecule *i*, and they do not show any correlation with the experimental relative toxicity. The $I_{std,i}^{OSMSI}$ data points of the HOMO and HOMO−1 orbitals are rather scattered against the experimental relative toxicity, and it is difficult to find correlation between the $I_{std,i}^{OSMSI}$ values and the experiment. In contrast to these observations, the $I_{std,i}^{OSMSI}$ values of the LUMO and LUMO+1 orbitals appear on the whole to monotonically decrease with the decrease in the experimental relative toxicity. The correlations of the $I_{std,i}^{OSMSI}$ values of the LUMO and LUMO+1 orbitals with the experiment are both reasonably well reproduced by a linear relationship, as can be seen by the linear regression lines in Figure 6. Here the Pearson correlation coefficients for linear regression analysis of the LUMO and LUMO+1 data are rather large and they are both 0.80 and 0.75, respectively, so the observed linear relationships are reliable to a reasonable degree. Interestingly, slopes of the linear regression lines for the LUMO and LUMO+1 data are comparable to each other. Nevertheless, such a relationship with the experimental relative toxicity is not observed for another unoccupied MO with a higher energy, LUMO+2. In addition, it may be worthwhile to note that if the experimental relative toxicity is calculated using either the intraperitoneal or oral LD_50_ values in Table 1, the $I_{std,i}^{OSMSI}\;$ values of the LUMO and LUMO+1 orbitals do not show any noticeable relationship, as we expected and described in Section 2.1, although the results are not depicted here.

At this stage, one may be curious about how the $I_{std,i}^{OSMSI}$ values of the LUMO and LUMO+1 orbitals are affected by the choice of the standard reference molecule and the level of theoretical calculations. Regarding the former case, if the standard reference molecule is changed, for instance, to *o*-isopropoxyphenyl-methylcarbamates having the third largest toxicity strength, the $I_{std,i}^{OSMSI}$ values of the LUMO and LUMO+1 orbitals exhibit essentially an inverted V-shape dependence, while it always peaks at the experimental relative toxicity corresponding to the chosen standard reference molecule. This is completely natural, because the $I_{std,i}^{OSMSI}$ value is defined as an index of how much $\rho_{i}\left(p_{k}\right)$ of an MO of the test molecule is similar to $\rho_{std}\left(p_{j}\right)$ of the corresponding MO of the standard reference molecule. On the other hand, regarding the latter case, it has been found that the presence of the observed correlations of the $I_{std,i}^{OSMSI}$ values of the LUMO and LUMO+1 orbitals with the experiment do not depend upon the level of theoretical calculations. For instance, Hartree–Fock calculations using the same 6-31G** basis set provide essentially the same dependence as the DFT B3LYP/6-31G** calculations, while the Pearson correlation coefficients for linear regression analysis of the LUMO and LUMO+1 data become slightly changed to 0.85 and 0.73, respectively. Likewise, we have tested the validity of consideration of the rotamer distribution over the C-C-O-C dihedral angle. For instance, in a case when we consider only the most stable rotamer for each methylcarbamate derivative, the DFT B3LYP/6-31G** calculations provide smaller Pearson correlation coefficients of 0.75 and 0.69 for the LUMO and LUMO+1 data, respectively. Consideration of the rotamer distribution is thus justified. All these observations suggest that spatial shapes of the LUMO and LUMO+1 orbitals have a direct link with the intravenous LD_50_ values of the methylcarbamate derivatives to some extent.

### 3.4. OS-MSI and Drug Reaction Mechansim

**Figure 7:**
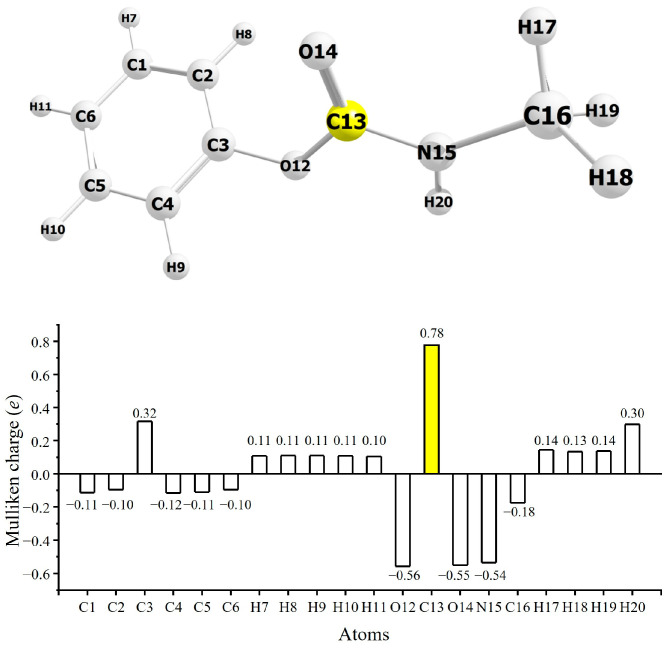
Mulliken charge distribution of phenyl methylcarbamate at the dihedral angle of 58.7° in water solvent.

A vital clue for understanding the above-made observations can be found in the drug reaction mechanism of the methylcarbamate derivatives. In the proposed reaction mechanism [15], the electrophilic carbonyl carbon atom of a carbamate derivative is supposed to react with a serine of the active site in the AChE enzyme. We have thus checked the electrophilicity of the carbonyl carbon atom of the methylcarbamate derivatives by using the Mulliken charge analysis [28,29] with the Gaussian 16 package at the DFT B3LYP/6-31G** level [21]. An example of the results is shown in Figure 7, which displays a Mulliken charge distribution obtained for the most stable rotamer of phenyl methylcarbamate at the dihedral angle of 58.7° in water solvent. Since the Mulliken charge, given in units of the elementary charge *e*, of an atom is calculated by subtracting the so-called gross population from the number of the atom′s valence electrons [29], a positive Mulliken charge means that the atom is electrophilic. It is clear from Figure 7 that the carbonyl carbon atom of phenyl methylcarbamate is highly electrophilic, supporting the proposed reaction mechanism. The high electrophilicity of the carbonyl carbon atom has also been confirmed for other methylcarbamate derivatives in the same way.

By considering the high electrophilicity of the carbonyl carbon atom together with the frontier orbital theory [18], one can conclude that the LUMO orbital is the frontier orbital in the drug reaction of the methylcarbamate derivatives. By keeping in mind that the LUMO+1 orbital is energetically very close to the LUMO orbital, as can be seen from Figure 5, one may additionally conclude that the LUMO+1 orbital also plays an equally crucial role in the drug reaction as another frontier orbital and this is consistent with the observation that the slopes of the linear regression lines for the LUMO and LUMO+1 data are almost the same as each other. When the same thinking is applied to the LUMO+2 orbital, it turns out that the energy of the orbital is too high to be a frontier orbital that gains energy through the drug reaction. On the other hand, the observation for the HOMO and HOMO−1 orbitals can be understood by considering the proposed reaction mechanism [15] which requires the methylcarbamate derivatives to be electrophile; all the occupied MOs, including the HOMO and HOMO−1 orbitals cannot be the frontier orbital but are spectators in the reaction mechanism. This is the reason why the traditional $I_{std,i}^{MSI}$ values associated with total electron momentum density remain unaltered with the change in the test molecule and no correlation of the $I_{std,i}^{OSMSI}$ values with the experimental relative toxicity is observed for the HOMO and HOMO−1 orbitals.

## 4. Summary

We reported on the computational investigation on the reachability of the MSI approach for predicting relative drug strength of the methylcarbamate derivatives, including the OS-MSI method that we proposed in the present study. It has been shown that the traditional MSI values associated with total electron momentum density as well as the OS-MSI values for the HOMO and HOMO−1 orbitals do not show correlation with the experimental relative toxicity. However, it has been observed and found that the OS-MSI values for the LUMO and LUMO+1 orbitals exhibit a strong correlation with the experiment, with the Pearson correlation coefficients of 0.80 and 0.75 in the linear regression analysis. Furthermore, the reason why only the LUMO and LUMO+1 orbitals are linked to the experimental relative toxicity is discussed in relation to the drug reaction mechanism.

However, there is ample room for testing the reachability of the MSI approach. In particular, the OS-MSI method should be tested preferentially by drug compounds having a nucleophilic nature so that their HOMO orbitals would play a crucial role in the drug reaction. In this regard, we started this test with the experimental antioxidant activity of flavonoid compounds [30], for which a first-principle theoretical study predicts that the HOMO orbital plays an important role in the reaction [31]. Our preliminary results on flavonoids clearly indicate that the OS-MSI values for the HOMO orbital show a strong correlation with the experimental relative activity. From the findings of the preliminary results, we have gained increased confidence that the MSI approach, in particular the OS-MSI method, has the potential ability not only to predict relative drug strength but also to identify the key MOs that govern the drug reaction mechanism.

Finally, it may be worthwhile to compare the MSI approach with the existing quantitative structure–activity relationship (QSAR) approach [32,33]. The QSAR approach, often combined with machine learning techniques, is known as a very powerful method to obtain quantitative prediction of the potency of biological activity, by relating a set of many physicochemical molecular properties such as energy and polarizability. On the other hand, although the MSI approach has proven its potential ability, it is still limited and far from quantitative prediction. In this regard, future efforts are required for practical use of the OS-MSI method in drug discovery.

## Figures and Tables

**Table 1:** Experimental LD_50_ values (from Ref. [14]) of methylcarbamate derivatives.

Derivatives	LD_50_ (mol/kg) ^(a)^		
	Intravenous ^(b)^	Intraperitoneal ^(b)^	Oral ^(b)^
phenyl	9.00 × 10^−5^	2.36 × 10^−3^	3.57 × 10^−3^
*m*-isopropylphenyl	1.63 × 10^−5^	7.35 × 10^−5^	2.03 × 10^−4^
*m*-cym-5-yl	2.56 × 10^−5^	1.31 × 10^−4^	6.03 × 10^−4^
*o*-isopropoxyphenyl	5.07 × 10^−5^	1.43 × 10^−4^	4.78 × 10^−4^
4-benzotheinyl	1.20 × 10^−4^	1.97 × 10^−4^	1.62 × 10^−3^
3,5-diisopropylphenyl	1.26 × 10^−4^	1.13 × 10^−3^	4.25 × 10^−3^
1-naphtyl	2.08 × 10^−4^	9.94 × 10^−4^	1.24 × 10^−3^
*o*-isopropylphenyl	3.42 × 10^−4^	7.35 × 10^−4^	1.94 × 10^−3^

^(a)^ LD_50_ is the amount of a toxic agent that kills 50% (one half) of a population of test animals. Here the amount is expressed in mol/kg. ^(b)^ LD_50_ values by three routes of administration (intravenous, intraperitoneal, and oral injections).

**Table 2:** Weight factors for methylcarbamate derivatives in water solvent at ten dihedral angles θ.

phenyl										
θ	28.7°	58.7°	88.7°	125.6°	148.7°	208.7°	234.4°	268.7°	301.3°	328.7°
weight	4.9%	15.1%	9.6%	16.1%	4.3%	4.3%	15.8%	9.6%	15.3%	5.0%
*m*-isopropylphenyl										
θ	31.4°	61.4°	91.4°	125.2°	151.4°	211.4°	234.8°	271.4°	298.6°	321.4°
weight	5.2%	14.6%	9.7%	16.7%	4.2%	4.2%	16.7%	10.7%	11.4%	6.7%
*m*-cym-5-yl										
θ	36.5°	66.5°	96.5°	115.4°	146.5°	216.5°	239.1°	266.5°	293.5°	326.5°
weight	6.8%	16.6%	4.2%	14.8%	7.6%	6.4%	14.3%	7.2%	15.4%	6.8%
*o*-isopropoxyphenyl										
θ	64.4°	74.4°	84.4°	94.4°	124.4°	235.6°	265.6°	275.6°	285.6°	295.6°
weight	0.0%	28.4%	15.7%	0.0%	6.1%	6.0%	0.0%	16.9%	26.9%	0.0%
4-benzothienyl										
θ	64.6°	84.6°	104.4°	125.4°	154.6°	204.6°	234.6°	254.6°	274.6°	294.6°
weight	1.9%	17.9%	1.0%	22.8%	6.3%	6.2%	23.3%	0.2%	18.1%	2.3%
3,5-diisopropylphenyl										
θ	33.7°	63.7°	93.7°	120.6°	143.7°	213.7°	239.4°	273.7°	296.3°	323.7°
weight	6.0%	15.5%	8.5%	13.0%	7.1%	6.0%	15.6%	9.0%	12.5%	6.9%
1-naphthyl										
θ	42.4°	92.4°	117.6°	142.4°	162.4°	192.4°	212.4°	242.4°	272.4°	302.4°
weight	0.0%	19.8%	14.7%	14.3%	0.0%	0.0%	12.3%	21.0%	17.8%	0.0%
*o*-isopropylphenyl										
θ	62.2°	72.2°	92.2°	112.2°	142.2°	207.8°	237.8°	267.8°	287.8°	297.8°
weight	0.0%	0.0%	34.7%	7.5%	6.8%	3.0%	10.3%	36.0%	0.0%	0.0%

## Data Availability

The data that support the findings of this study are available from the corresponding author upon reasonable request.

## Supplementary Materials

The following supporting information can be downloaded at: https://www.mdpi.com/article/10.3390/molecules30142947/s1, the OS-MSI calculations information. References [7,21,34] are cited in the supplementary materials.
