# Supplementary material for: Observation of a Relationship Between Orbital-Specific Molecular Similarity Index and Toxicity of Methylcarbamate Derivatives
Source: Molecules. 2025 Jul 12;30(14):2947. doi: 10.3390/molecules30142947 (PMC12299121; doi:10.3390/molecules30142947)
Supplement: Supplementary file 1 [file molecules-30-02947-s001.zip › molecules-3732151-supplementary.pdf]

Supporting information for

Observation of a relationship between orbital-specific molecular similarity index and toxicity of methylcarbamate derivatives

Sihan Long, Yuuki Onitsuka, Soichiro Nagao and Masahiko Takahashi *

Institute of Multidisciplinary Research for Advanced Materials, Tohoku University, Sendai 980-8577, Japan
* Correspondence: masahiko@tohoku.ac.jp

1. Molecular similarity index calculation workflow

The molecular similarity index (MSI) and orbital-specific molecular similarity index (OS-MSI) values are calculated in this study according to workflow steps in Figure S1.

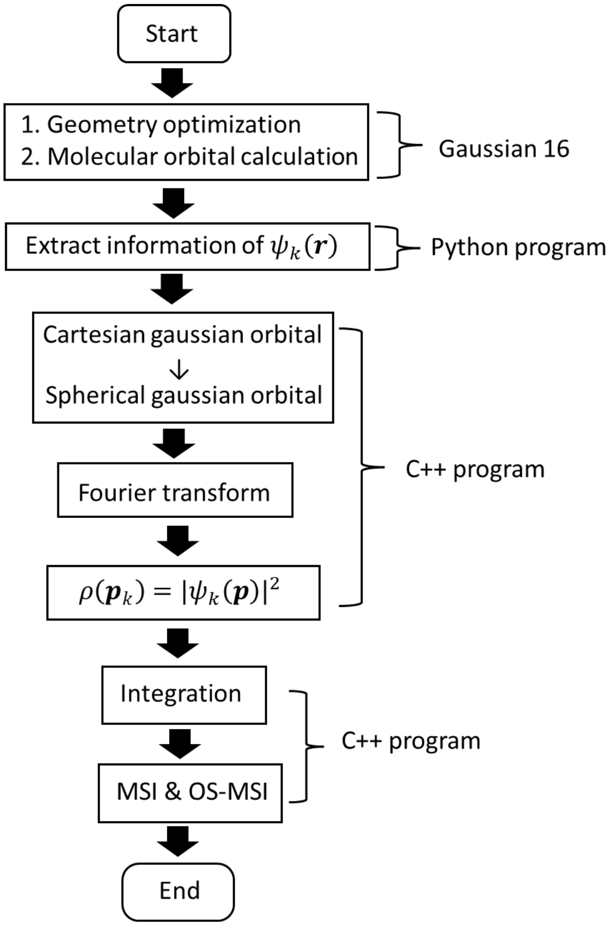

Figure S1. MSI & OS-MSI calculation workflow.

2. Quantum chemistry calculation

Geometry optimization of a molecule with *N* atoms and calculation of molecular orbitals (MOs) can be made using the Gaussian 16 package [1]. In a Gaussian output file, the *k*-th MO, $\psi_k(\boldsymbol{r})$, is calculated as a linear combination of Gaussian-type atomic orbitals (AOs) in Cartesian form:

$$\psi_k(\boldsymbol{r}) = \sum_{A}^{N}\sum_{A}^{nA} C_{kAa}\phi_{Aa}(\boldsymbol{r} - \boldsymbol{R_a}), \quad \text{(S1)}$$

where a is the index of basis function $\phi_{Aa}(\boldsymbol{r})$ on atom A. $\boldsymbol{R_a}$ refers to the coordinate of atom A, and nA is the number of AOs used for the atom.

The momentum-space MO, $\psi_k(\boldsymbol{p})$, can be obtained by Fourier transform of $\psi_k(\boldsymbol{r})$:

$$\psi_k(\boldsymbol{p}) = (2\pi)^{-\frac{3}{2}}\int \mathrm{d}\boldsymbol{r}\exp(-i\boldsymbol{p}\cdot\boldsymbol{r})\psi_k(\boldsymbol{r}). \quad \text{(S2)}$$

We use a home-made python program to extract all of the required information, such as atomic positions and expansion coefficients and quantum numbers of AOs.

2. Fourier transform

We have developed a C++ program to convert the position-space MO, $\psi_k(\boldsymbol{r})$, to the momentum-space, MO $\psi_k(\boldsymbol{p})$. Generally speaking, a position-space AO, $\phi_{nlm}(\boldsymbol{r})$**,** is defined in spherical coordinate form with quantum numbers *n, l, m*:

$$\phi_{nlm}(\boldsymbol{r}) = f_{nl}(r)Y_{lm}(\Omega), \quad \text{(S3)}$$

where the $f_{nl}(r)$ and $Y_{lm}(\Omega)$ denotes radial and angular parts, respectively.

Here

$$f_{nl}(r) = B(n,\alpha)r^{n-1}e^{-\alpha r^2}, \quad \text{(S4)}$$

with Gamma function $B(n,\alpha)$ defined by

$$B(n,\alpha) = \left(\frac{2(2\alpha)^{n+\frac{1}{2}}}{\Gamma\left(n+\frac{1}{2}\right)}\right)^{\frac{1}{2}}, n-1 > 1 > 0. \quad \text{(S5)}$$

The e^{-ipr} term in Equation (S2) can be expressed by

$$e^{-i\boldsymbol{pr}} = 4\pi \sum_{l}^{\infty} \sum_{m=-l}^{l} (-i)^l j_l(pr) Y_{lm}^{*}(\theta,\phi) Y_{lm}(\theta,\phi)\,. \tag{S6}$$

The momentum-space AO, $\phi_{nlm}(\boldsymbol{p})$, is thus given by

$$\phi_{nlm}(\boldsymbol{p}) = N_{nlm} u_{nl}(p) Y_{lm}(\Omega). \tag{S7}$$

The radial part of $\phi_{nlm}(\boldsymbol{p})$**,** $u_{nl}(p)$, is obtained by Hankel integral:

$$\begin{aligned} u_{nl}(p) &= (-i)^l \left(\frac{2}{\pi}\right)^{\frac{1}{2}} \int_0^{\infty} j_l(pr) f_{nl}(r) r^2 dr \\ &= (-i)^l p^{-1} h_{nl}(p), \end{aligned} \tag{S8}$$

$$h_{nl}(p) = p \left(\frac{2}{\pi}\right)^{\frac{1}{2}} \int_0^{\infty} j_l(pr) f_{nl}(r) r^2 dr\,. \tag{S9}$$

$h_{nl}(p)$ can be analytically calculated when $n - l$ is odd [2]. For instance, in a case when $l = 0$ and n is odd, $h_{nl}(p)$ is given by

$$h_{nl}(p) = B(n,\alpha) \frac{(-1)^V e^{-z}}{2^{n+\frac{1}{2}} \alpha^{\frac{n+1}{2}}} H_n\left(z^{\frac{1}{2}}\right). \tag{S10}$$

Here $H_n(x)$ is the Hermite polynomial and it is given by

$$H_n(x) = (-1)^n e^x \frac{d^n}{dx^n}\left(e^{-x^2}\right), \text{with } z = \frac{p^2}{4\alpha}. \tag{S11}$$

It should be noted that in the Gaussian 16 package, all the AOs satisfy the requirement of $l = 0$ and odd n, so the momentum-space MO is analytically obtained as a linear combination of Gaussian-type AOs, $\phi_{nlm}(\boldsymbol{p})$.

$$\psi_k(\boldsymbol{p}) = \sum_{a}^{N} \exp\left(-ip \cdot \boldsymbol{R_a}\right) \sum_{b}^{na} C_{jab} \phi_{ab}(\boldsymbol{p}) \tag{S12}$$

The electron momentum density, $\rho(\boldsymbol{p}_k)$, is obtained by taking the absolute square of k-th MO, $\psi_k(\boldsymbol{p})$:

$$\rho(\boldsymbol{p}_k) = |\psi_k(\boldsymbol{p})|^2. \tag{S13}$$

The total electron momentum density of a test molecule i is obtained by summing up the electron momentum density over all the occupied MOs:

$$\rho_i(\boldsymbol{p}) = \sum_k \rho_i(\boldsymbol{p}_k) \tag{S14}$$

3. MSI and OS-MSI calculations

We have developed a C++ program to calculate the MSI and OS-MSI values between a reference molecule *std* and a test molecule *i*, according to Equations (S15) and (S16):

$$I_{std,i}^{\mathrm{MSI}}(n) = \frac{2\int p^n\left[\sum_{\boldsymbol{j}}\rho_{std}(\boldsymbol{p}_j)\right]\left[\sum_{\boldsymbol{k}}\rho_i(\boldsymbol{p}_k)\right]\mathrm{d}\boldsymbol{p}}{\int p^n\left[\sum_{\boldsymbol{j}}\rho_{std}(\boldsymbol{p}_j)\right]^2\mathrm{d}\boldsymbol{p} + \int p^n\left[\sum_{\boldsymbol{k}}\rho_i(\boldsymbol{p}_k)\right]^2\mathrm{d}\boldsymbol{p}}, \tag{S15}$$

$$I_{std,i}^{\mathrm{OSMSI}} = \frac{2\int p^n\rho_{std}(\boldsymbol{p}_j)\rho_i(\boldsymbol{p}_k)\mathrm{d}\boldsymbol{p}}{\int p^n\left[\rho_{std}(\boldsymbol{p}_j)\right]^2\mathrm{d}\boldsymbol{p} + \int p^n\left[\rho_i(\boldsymbol{p}_k)\right]^2\mathrm{d}\boldsymbol{p}}. \tag{S16}$$

In the computation program, the integration steps for the three-dimensional electron momentum densities are defined by radial step Δp [a.u.], and angular steps $\Delta\theta$ [deg] and $\Delta\varphi$ [deg] as shown in Figure S2. To determine appropriate integral ranges and steps, we have checked convergency of MSI values by changing the integration steps.

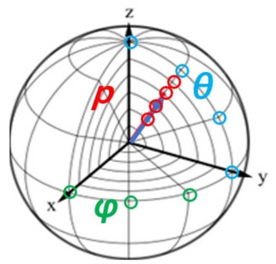

Figure S2. Radial and angular steps in MSI integrations.

As an example, we show dependence of the angular steps, $\Delta\theta$ and $\Delta\varphi$, on MSI values for H_2O-CH_3OCH_3 and H_2S-CH_3SCH_3 in Figure S3. It can be seen that the change in the MSI values is minimal against the change of the angular steps. We thus take the angular step of 5° for both $\Delta\theta$ and $\Delta\varphi$, to save computational cost.

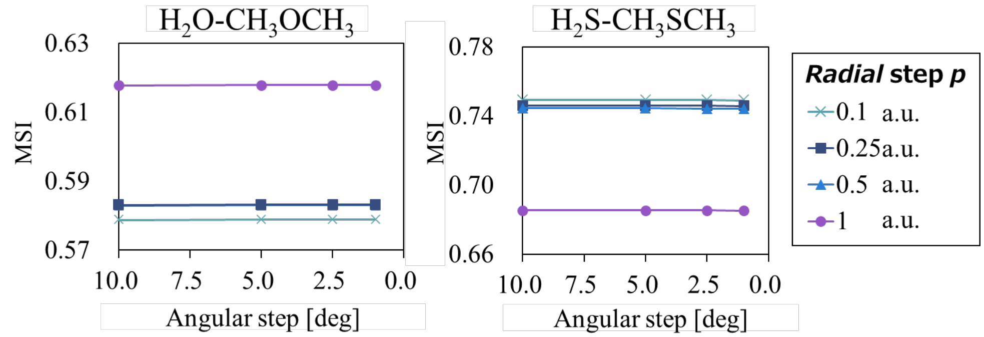

Figure S3. Dependence of the angular steps, $\Delta\theta$ and $\Delta\varphi$, on MSI values.

In contrast, dependence of the radial step, Δp, is substantially large, as can be seen in Figure S4 which plots MSI values obtained while keeping the angular step of 5° for both $\Delta\theta$ and $\Delta\varphi$. We thus take the radial step of 0.1 a.u. to achieve reliable accuracy.

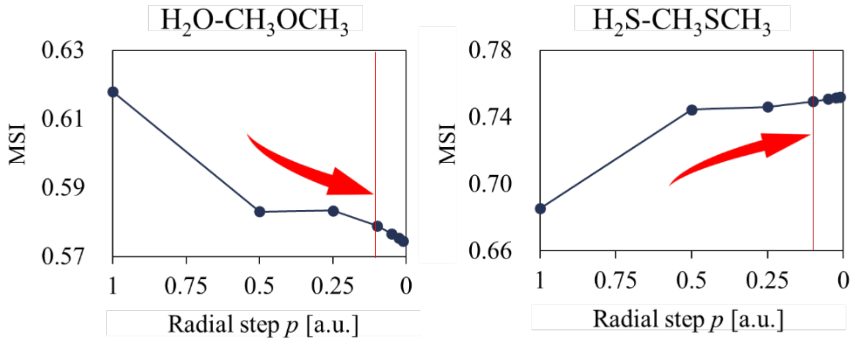

Figure S4. Dependence of the radial step, Δp, on MSI values.

The remaining task to calculate MSI is to determine an appropriate integral range in momentum space. As an example of our test calculations, we show in Figure S5 the calculated radial electron momentum density $p^2\rho(p)$ for the five MOs of H_2O. It can be seen that the density distributions of all the orbitals

approach zero at around 20 a.u. However, to ensure computational rigor for larger molecules, we calculate MSI value over an integral range of up to 30 a.u.

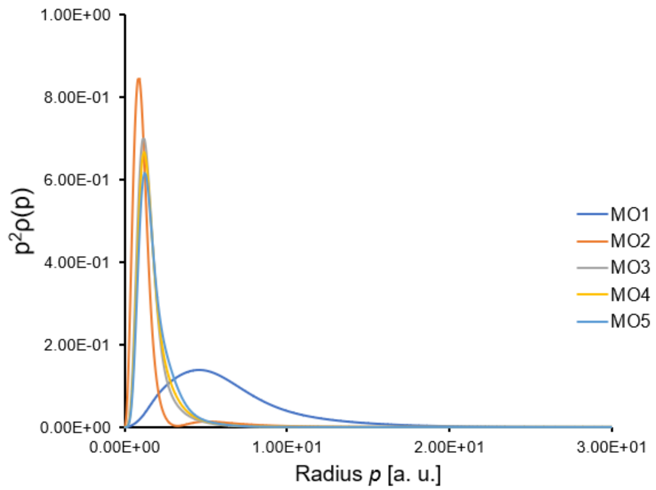

Figure S5. Radial electron momentum density of H_2O.

Finally, we have checked our home-made program by comparing the calculated results with the data in literature [3]. Figure S6 shows MSI results of the comparison made for five different molecules. It can be seen that differences in MSI values between our calculated results and the literature [3] are all within 0.6%.

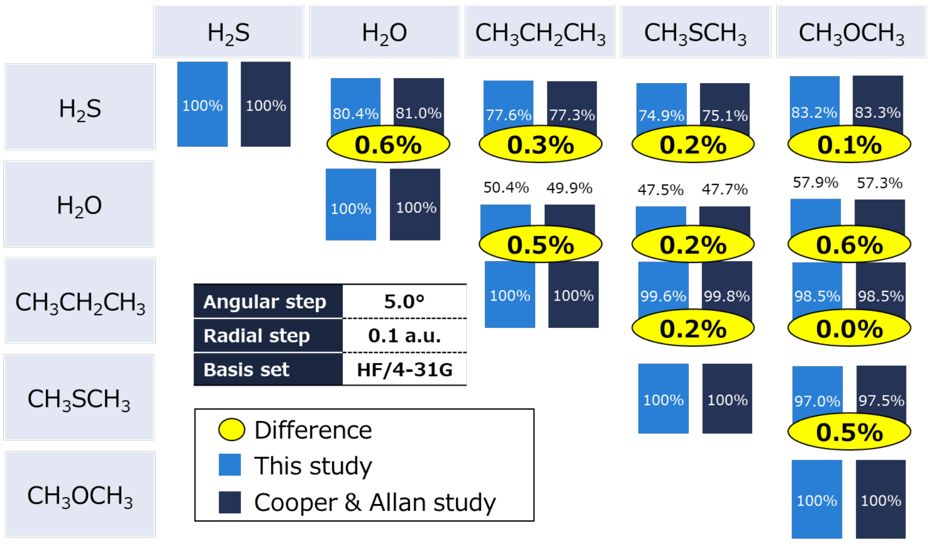

Figure S6. Comparison of MSI values between ours and literature [3].

4. Computational cost

Finally, we mention about computational cost. The present work conducts all the calculations using a workstation with the following computer configuration.

Operating System	Ubuntu 24.04.2 LTS
Processor	Intel®Xeon® Gold 6240R x 96
Memory	64 GB

Computational cost is largely dependent on the number of electrons in the molecule. However, as an example, we show computational cost required for the calculations for 4-benzothienyl-methylcarbamate.

Geometry optimization	1h34min
Molecular orbital calculation (one rotamer)	1min
Electron momentum density (one MO)	10min

References

[1] Frisch, M.J.; Trucks, G.W.; Schlegel, H.B.; Scuseria, G.E.; Robb, M.A.; Cheeseman, J.R.; Scalmani, G.; Barone, V.; Petersson, G.A.; Nakatsuji, H.; et al. Gaussian 16 Revision 16.A.03; Wallingford CT. Inc.: Wallingford, CT, USA, 2016.

[2] Kaijser, P.; Smith Jr, V. H. Evaluation of momentum distributions and Compton profiles for atomic and molecular systems. *Adv. Quantum Chem.* **1977**, *10*, 37-76.

[3] Cooper, D. L.; Allan, N. L. A novel approach to molecular similarity. *J. Comp-Aided Mol Des.* **1989**, *3*, 253-259.
